# Physiological and ecological characteristics and reproductive responses of* Phragmites australis* to dry-wet conditions in inland saline marshes of Northeast China

**DOI:** 10.7717/peerj.14269

**Published:** 2022-10-31

**Authors:** Cui Mingyang, Du Zhixin, Li Xiaoyu, Chen Junze

**Affiliations:** 1 Northeast Institute of Geography and Agroecology, Chinese Academy of Sciences, Changchun, China; 2 Yanbian University, Yanji, China; 3 Northeast Normal University, Changchun, China

**Keywords:** *Phragmites australis*, Hydrological variation, Root structure, Reproductive characteristics, Plant physiology

## Abstract

Inland saline marshes in northeastern China have unique soil characteristics and population distribution features. Hydrological change is a critical environmental factor causing wetland degradation and soil salinization in this region. The growth and reproductive responses of typical wetland plants to dry-wet alternations are essential for restoring inland saline marshes. A pot experiment was conducted to study the growth and reproductive responses of *Phragmites australis* populations to three hydrological treatments simulating drought degradation (drought), permanent inundation restoration (flooding), and seasonal inundation restoration (dry-wet). The species showed different growth and reproductive responses to the three treatments. After 120 d, the drought conditions induced a lower biomass, root length and root surface area of *P. australis*, but with higher root diameter, soluble sugar, and Na^+^ ion contents. Flooding and alternating dry-wet treatments induced the opposite responses. Alternating dry-wet treatments can be considered a better solution to effectively conserve water and meet the water needs of *P. australis* in the current growing season. The biomass under the alternating wet and dry treatment was the same as that under flooding, but the number of rhizome shoots was lower. The alternating dry-wet treatments was able to recover the growth of *P. australis* in the current season, but the potential for asexual reproduction of the species was insufficient.

## Introduction

Wetlands are some of the most biodiverse landscapes and essential natural resources. Wetland ecosystems are known as “nature’s kidneys” and “biological gene banks”, facilitating environmental regulation and species genetic conservation (Hu et al., 2017). Wetlands are classified as salt marshes, marsh wetlands, coastal wetlands, and mangroves, among others, of which salt marshes are the most essential types of wetland ecosystems (Gedan, Silliman & Bertness, 2009). The salt marshes on the Western Songnen Plain, Northeast China, have unique soil characteristics and population distribution features, which are dominated by rhizomes and tufted grasses. The predominant species is *Phragmites australis*, accompanying by *Typha orientalis* and *Scirpus planiculmis* (Fan et al., 2016). These salt marshes play an important ecological protection function in Northeast China. However, Since 1954, the marsh area on the Western Songnen Plain has decreased by 74%, while the saline area has been increasing 1.73 times from 401.48 × 10^3^ hm^2^ to 1,097.45 × 10^3^ hm^2^ (Li et al., 2008).

Against the background of a changing climate and human activities, the *P. australis* wetland ecosystem is facing serious problems such as area reduction, wetland fragmentation, pollution (deterioration of water quality), and overexploitation. Irrational cultivation and irrigation can cause salinization of the soil, which can lead to the degradation of saline marshes, a process known as secondary salinization of the land. Secondary salinization severely restricts the ecological and environmental protection function of the wetlands in the agricultural and livestock mosaic zone of northeastern China (Wang et al., 2004). Such degradation also reduces other ecological functions, such as the provision of waterfowl habitat and breeding grounds, regional climate regulation, water storage and flood diversion, and water purification, calling for the restoration of degraded saline wetlands (Zhao et al., 2022). Hydrology is an essential factor influencing the degradation of saline marshes and soil salinization. Water changes directly affect the accumulation of organic matter and nutrient cycling in saline marsh vegetation, altering primary productivity and species abundance (Niu et al., 2015). Dramatic water changes also affect the structure, nutrient cycling, and microbial composition of saline marsh soils, consequently influencing community formation and succession as well as ecosystem structure and function (Palanisamy & Chui, 2013).

Usually, wetland restoration techniques include three main categories: habitat restoration, biological restoration, and ecosystem structure and function restoration (Zhang & Wang, 2001). Because the objectives and strategies for restoring different types of degraded wetlands are different, the key technologies to be adopted are not the same. The current ecological restoration and reconstruction process of degraded saline wetlands is generally based on the characteristics of the saline wetland environment where “salt comes with water, and by salt goes with water”, by restoring surface runoff, increasing wetland water volume and elution saline components (Yang et al., 2009). Similarly, in the *P. australis* wetlands of the Western Songnen Plain, the ecological restoration process was to establish a surface water drainage and irrigation circulation system with the goal of drenching salinity through engineering methods to achieve habitat restoration and automatic biological restoration (Luo, Zhu & Sun, 2003).

*Phragmites australis* is a perennial grass that can reproduce clonally *via* rhizomes. It is ecologically adaptable and highly resilient (Packer et al., 2017). As a typical wetland plant, *P. australis* is a dominant and established species in inland salt marshes of the Western Songnen Plain, absorbing salt and purifying the water (Guan et al., 2017). The pH, conductivity, and major salt ion content of saline soils showed a decreasing trend during *P. australis* growing season, in the western Songnen Plain (Qiu, 2014). The wilting and decaying *P. australis* are also effective in reducing salinity and increasing organic matter content in saline soils (Sun et al., 2012). The *P. australis* wither and decay into humus, which can increase the organic matter content of saline soils and create chemical changes within the saline soils to neutralize the salts, thus reducing the salt content of saline soils. This species exhibits different growth and physiological responses to hydrological changes. Prolonged periods of severe drought reduce *P. australis* biomass, leading to a smaller tolerance range of it to environmental factors and accelerating the decline of *P. australis* populations (Ye et al., 1998). Drought can also inhibit physiological processes in *P. australis*, resulting in a reduced ability to tolerate increased light radiation intensity and relative air humidity; the species can limit its photosynthesis and biomass reduction by reducing water consumption *via* restricting stomata opening (Li et al., 2017). During flooding, reduced gas exchange between soil and air can lead to a lack of oxygen in the soil environment, leaving the ground in an anaerobic environment, reducing the soil redox potential, and affecting nutrient uptake by plant roots (Steffens et al., 2005).

*Phragmites australis* responds to anoxic conditions by forming aeration tissues and aerial roots. Its deep and extensive root system and reproduction characteristics also enhance its adaptation to environmental disturbances, making it a dominant species in the study area (Song, 2021). Alterations in wet and dry conditions change the allocation of *P. australis* biomass. Under drought conditions, the ratio of belowground to aboveground biomass increases; *P. australis* allocates more of its biomass to belowground organs, thereby facilitating water uptake (Engloner, 2009). In contrast, under flood conditions, *P. australis* reduces the root system to reduce oxygen consumption while increasing the proportion of biomass allocated to the foliage, thereby increasing its contact area with air to improve carbon dioxide uptake (Vretare et al., 2001). Whilst previous studies have mainly focused on short-term simulated controls to study the responses of individual plant seedlings to habitat changes, experiments throughout the entire growing season are scarce, particularly considering root growth and clonal reproduction responses of *P. australis* to hydrological changes in saline environments.

To study the resilience of *P. australis* populations and to develop effective hydrological management strategies under saline soil conditions, *P. australis* communities in highly saline soils on the Western Songnen Plain were selected for this study. A pot experiment was used to simulate three hydrological characteristics: drought, flooding, and alternating dry-wet conditions, and to compare and analyze the characteristics of changes in biomass, root structure, clonal reproduction, photosynthetic physiology and metabolic physiology of *P. australis* under hydrological changes to study the physiological and ecological processes and reproductive responses of *P. australis* populations to water changes. The results provide a scientific basis for the conservation, restoration, and management of saline marshes in the context of global change and facilitate the development of strategies to enhance the ecological functions of vulnerable wetlands.

## Material and methods

### Experimental materials

**Figure 1:**
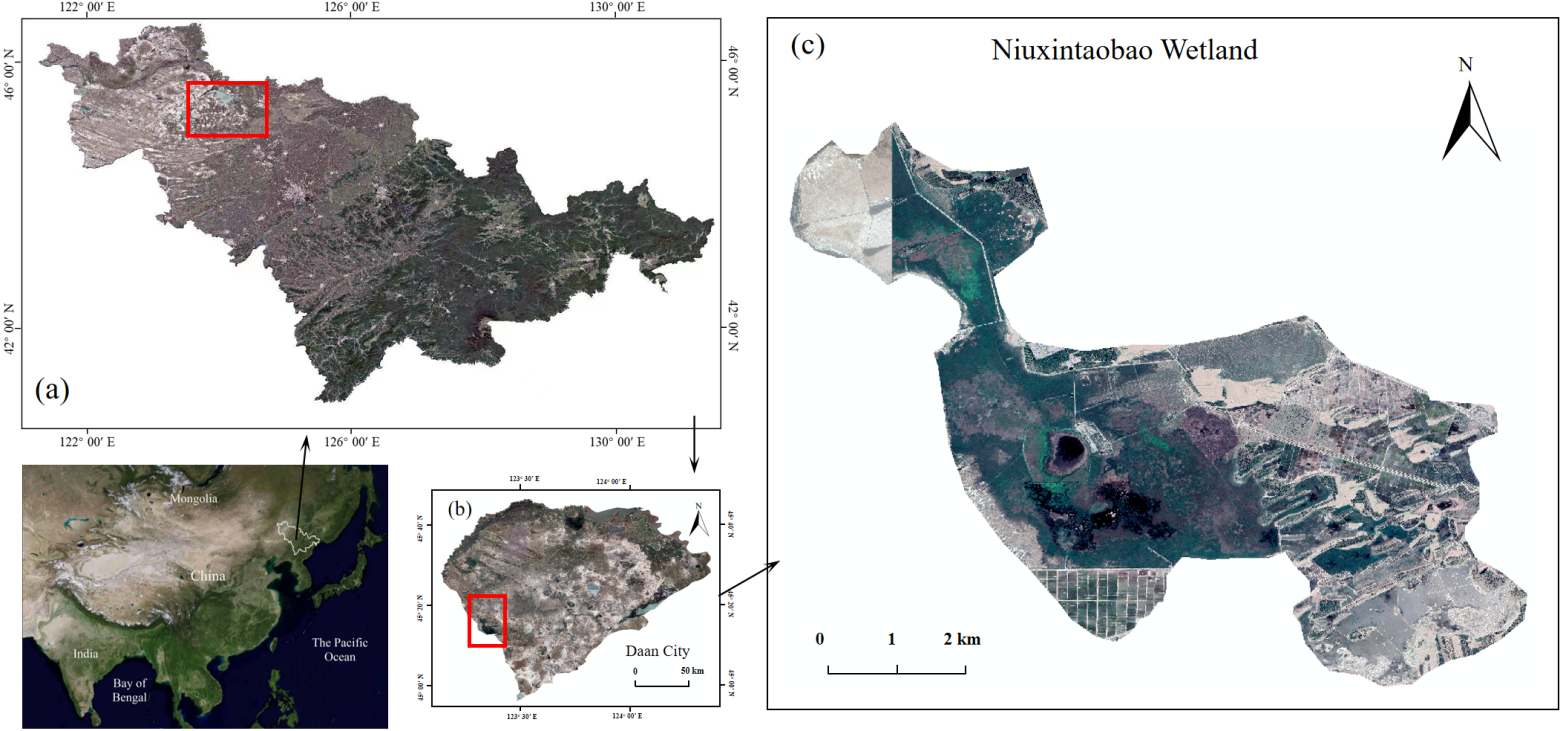
*Phragmites australis* wetlands in Western Songnen Plain of Northeast China. (A) Jilin Province in Northeast China, (B) Da’an City in West of Jilin Province, (C) Niuxintaobao Marsh for producing *P. australis*. The site map was generated by ArcGIS 10.2.

The *P. australis* and saline soils in the experiments were obtained from Niuxintaobao (Northeast Institute of Geography and Agroecology, Chinese Academy of Sciences—Saline Wetland Ecological Research Station of Western Songnen Plain, Da’an City, Jilin Province), with the geographical coordinates 45°14′N, 123°21′E and a total area of 4,200 hm^2^ (Fig. 1). Rhizome buds were dug out in early May 2019 after *P. australis* had returned to green; saline soil, seriously degraded (pH ≈10.4, EC ≈1,181 µs/cm), was sampled for the simulation experiment. Plant buds and soils were taken to the greenhouse and cultured to be used in the pot experiments. Table 1 shows the analyzed soil parameters, which is the soil quality status of seriously degraded *P. australis* marshes.

### Pot experimental design

**Figure 2:**
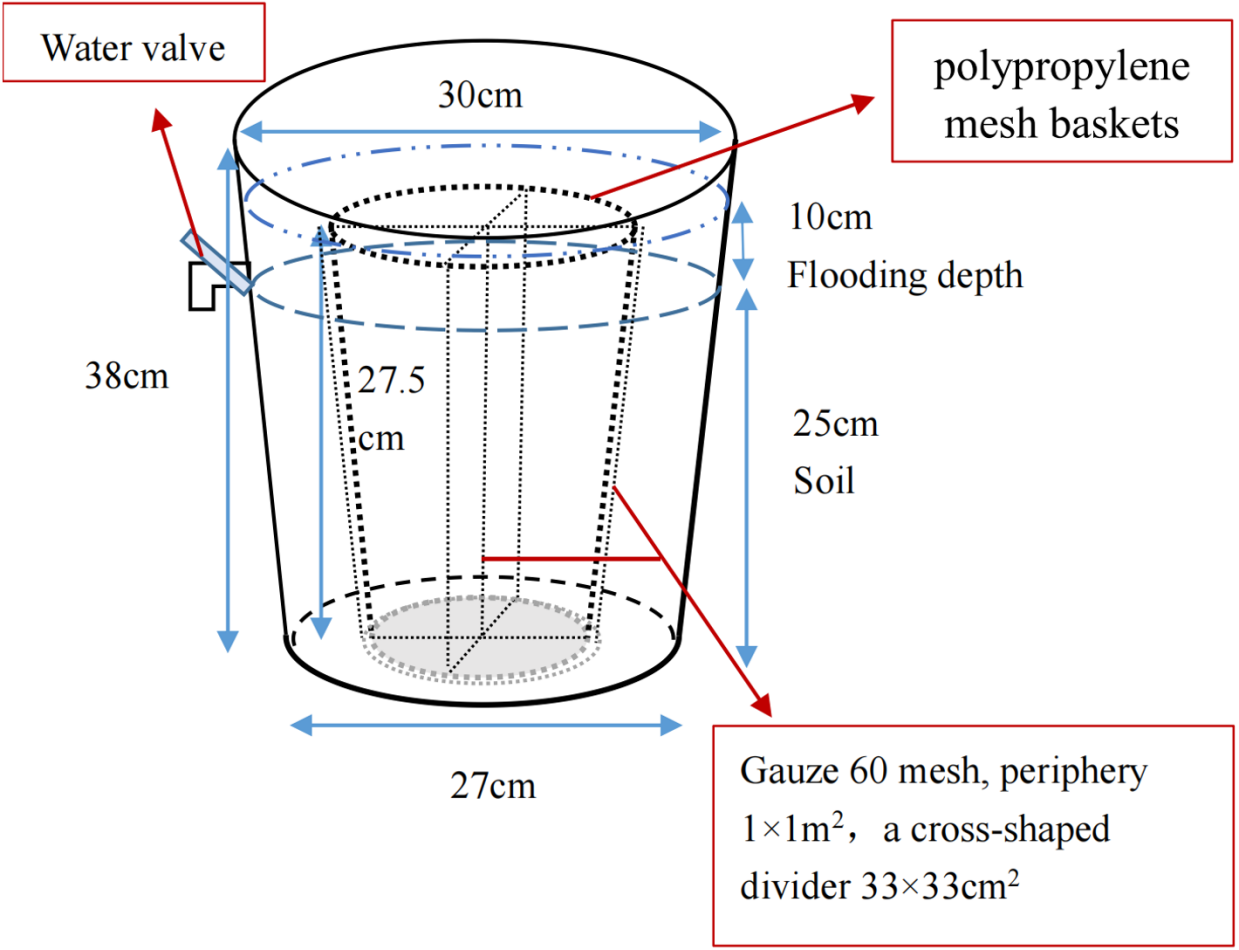
Potted plant pattern diagram.

The pot experiment was performed to simulate the restoration of saline soil to marsh soil in a greenhouse at the Northeast Institute of Geography and Agriculture, Chinese Academy of Sciences. Briefly, the saline soil was broken up into uniform particles and mixed thoroughly. Overall, 36 plastic containers (top diameter 30 cm, bottom diameter 27 cm, height 38 cm) and 36 polypropylene mesh baskets (maximum diameter 23.5 cm, base diameter 18.5 cm, height 27.5 cm) were prepared; gauze (60 mesh) was used to divide the baskets into four parts for calculating the growth and bud reproduction per plant. The homogeneous saline soil was packed into containers and compacted until the soil surface was 13 cm to the top edge of the container and all pots had an equal weight (Fig. 2). After adding equal amounts of water, four *P. australis* seedlings were planted into each pot in mid-May. All planted seedlings had a height of 15 cm and were attached to a section of rhizome (rhizomes of similar length), ensuring a growing recovery period of 1 month. Over time, the moisture content gradually decreased to about 15%. Wet and dry treatments were carried out in mid-June. As *P. australis* roots can grow extensively, the built-in net basket was designed to facilitate the collection of inter-and non-inter-root soil at a later stage, and a cross-shaped divider was added to the net basket to prevent roots from growing entangled with each other.

The experiment was set up with three treatments, namely drought (water content of about 15%, D), permanent flooding (10 cm water layer, W), and alternating dry-wet (drought-flood-drought-flood, DW), with 12 replications per treatment. The soil moisture of saline soil was 15% after we weighed and calculated. Thus, the drought level was set 35–40% of the field capacity to fit for the 15% of soil moisture, and flooding was set as a 10 cm water table over the soil surface. The alternating dry-wet treatment was set with an interval of 30 days, with the first drought phase containing about 15% water and the second drought phase draining the soil surface water, naturally falling dry for 30 d. The water level is always kept 10 cm above the soil surface when re-watering after each drought. All treatments were at 30-d intervals. The experimental period was 120 d for each treatment, measurements were taken and sampled at 30, 60, 90, and 120 days, respectively, and three pots were used for destructive sampling for each treatment at each sampling, for a total of nine pots at the same sampling time.

**Table 1:** Soil basic nutrients in test ground.

**pH**	**Organic matter**	**Electric conductivity**	**Total nitrogen**	**Total phosphorus**	**Total potassium**
	**%**	**µs/cm**	**g/kg**	**g/kg**	**g/kg**
10.44	0.60	734	0.19	0.31	24.02

### Measurement of photosynthetic physiology in *P. australis*

Five fully expanded new leaves were selected from each pot (the third leaf from the top of the plant) to determine net photosynthetic rate, stomatal conductance, intercellular CO_2_ concentration, and transpiration rate, using a LI6400XT portable photosynthesis apparatus (LI-6400XT, Li-Cor, Inc., Lincoln, NE, USA). Because the photosynthesis rates are highest at midday, which can cause the leaf stomata to close and affect the test results, and in the afternoon, when the light intensity decreases, the measurements were performed between 9:00 and 11:00 am (full sunlight).

### Measurement of *P. australis* growth and reproduction

After measuring the photosynthetic activity of *P. australis*, the above- and belowground parts were harvested separately. Stems and leaves were separated to dry at 65 °C to constant weight, and the biomass was recorded. By removing the mesh basket before collecting the roots and taking out the plant separator mesh, the roots of each plant were separated and root breakage was avoided. The rhizome buds and shoot tillers produced by each transplanted *P. australis* were counted, and some roots were bagged and frozen for root structure analysis. The remaining roots were dried to determine the individual root biomass and calculate the water content and root/shoot ratio.

The root system was scanned using a double-sided light source scanning system (EPSON Expression 1640XL, EPSON, Los Alamitos, CA, USA) and analyzed using digital software (WinRHIZO-2004a) to obtain morphological indicators such as root length, root surface area, and root diameter and to calculate the specific root length and root surface area, using the following equations:

\begin{eqnarray*}\text{Water content} (\text{%})& = \frac{\text{Fresh weight}-\text{Dry weight}}{\text{Fresh weight}} \times 100\text{%} \end{eqnarray*}

\begin{eqnarray*}\text{Roots}/\text{Shoots}& =\text{Dry weight of roots}/\text{Dry weight of shoots} \end{eqnarray*}

\begin{eqnarray*}\text{Specific root length}& =\text{Root length}/\text{Root biomass} \end{eqnarray*}

\begin{eqnarray*}\text{Specific root surface area}& =\text{Root surface area}/\text{Root biomass}. \end{eqnarray*}

### Determination of metabolic physiology in *P. australis*

Dried stems and leaves were powdered using a ball mill to measure soluble sugar, proline, and cation contents. The soluble sugars were determined using the anthrone colorimetric method (UV spectrophotometer, Hach, Loveland, CO, USA). The Na^+^ and K^+^ contents were measured by flame photometry, and the Ca^2+^ and Mg^2+^ contents were determined *via* complex titration. Table 2 summarizes the meaning of the different physiological parameters measured in the study.

**Table 2:** The meaning of the different physiological parameters measured in the study.

	**Parameters**	**Function**
*P. australis* growth and reproduction	Aboveground biomass	It directly reflects the growth of the plant
	Rhizome shoots Tiller seedlings	They are a sign of good or bad plant development
	Root biomass	It is an important indicator of the growth and development of the plant root system
	Root diameter Root length Root surface area	They reflect the adaptive characteristics of plants to different habitats
	Specific root length Specific root surface area	They reflect the root expansion capacity
Photosynthetic physiology in *P. australis*	Net photosynthetic rate	It reflects the amount of organic matter accumulated by the plant per unit time
	Transpiration rate	It reflects the degree of stomatal opening
	Intercellular CO_2_ concentration	It is the main factor affecting the change of photosynthetic rate
	Stomatal conductance	It is the main factor affecting photosynthesis, respiration and transpiration
Metabolic physiology in *P. australis*	Soluble sugars Proline	Plants maintain cellular osmotic pressure by increasing intracellular proline and soluble sugar content, thereby increasing resilience
	Cation ions	It is involved in regulating the physiological activities of plant cells

### Statistical analysis

One-way and two-way analysis of variance (ANOVA) were performed on the data using the SPSS 23.0 software (SPSS Inc, Chicago, IL, USA), with multiple comparisons using the least significant difference (LSD) at a significance level of 0.05. This experiment tested the assumption of homogeneity of variances (Levene’s) and the normal distribution. A transformation was necessary to satisfy the statistical requirement if the data were not normally distributed. Drawbar graphs were generated using the Origin 2019 software (Origin Lab Corporation, Northampton, MA, USA).

## Results

### Correlation study of different water treatments and growth time on various aspects of *P. australis*

The water treatment factor was not significant for proline in leaves and had a significant effect on the rest of the indicators. Development time had a non-significant effect on specific root length, root diameter and K^+^ in stems, and a significant effect on all other indicators. The interaction of water treatment and developmental time had a non-significant effect on specific root surface area, aboveground biomass and K^+^ in stems, while it had a significant effect on all other indicators (Table 3).

**Table 3:** Analysis of variance of water treatment, development time and interaction on different indicators.

	**Root length**	**Specific root length**	**Root surface area**	**Specific root surface area**	**Root diameter**	**Root biomass**	**Rhizome shoots**	**Tiller seedlings**	**Aboveground biomass**
Water treatment	0.000^***^	0.000^***^	0.000^***^	0.000^***^	0.000^***^	0.000^***^	0.000^***^	0.000^***^	0.000^***^
Development time	0.000^***^	0.304	0.000^***^	0.039^*^	0.735	0.000^***^	0.000^***^	0.000^***^	0.023^*^
Water treatment × Development time	0.000^***^	0.036^*^	0.000^***^	0.349	0.005^***^	0.000^***^	0.000^***^	0.020^**^	0.099

	**Net photosynthetic rate**	**Transpiration rate**	**Intercellular CO_2_ concentration**	**Stomatal conductance**
Water treatment	0.000^***^	0.000^***^	0.000^***^	0.000^***^
Development time	0.000^***^	0.000^***^	0.000^***^	0.000^***^
Water treatment × Development time	0.042^*^	0.000^***^	0.000^***^	0.000^***^

	**Soluble sugars in stems**	**Soluble sugars in leaves**	**Proline in stems**	**Proline in leaves**	**Na^+^ in leaves**	**K^+^ in leaves**	**Na+ in stems**	**K+ in stems**
Water treatment	0.000^***^	0.042^*^	0.000^***^	0.074	0.000^***^	0.018^*^	0.000^***^	0.000^***^
Development time	0.000^***^	0.000^***^	0.000^***^	0.000^***^	0.000^***^	0.000^***^	0.000^***^	0.329
Water treatment × Development time	0.000^***^	0.000^***^	0.000^***^	0.000^***^	0.000^***^	0.004^***^	0.001^***^	0.103

**Notes.**

* Significantly different at *p* < 0.05.

** Significantly different at *p* < 0.01.

*** Significantly different at *p* < 0.001.

### *P. australis* biomass and root/shoot ratio

**Figure 3:**
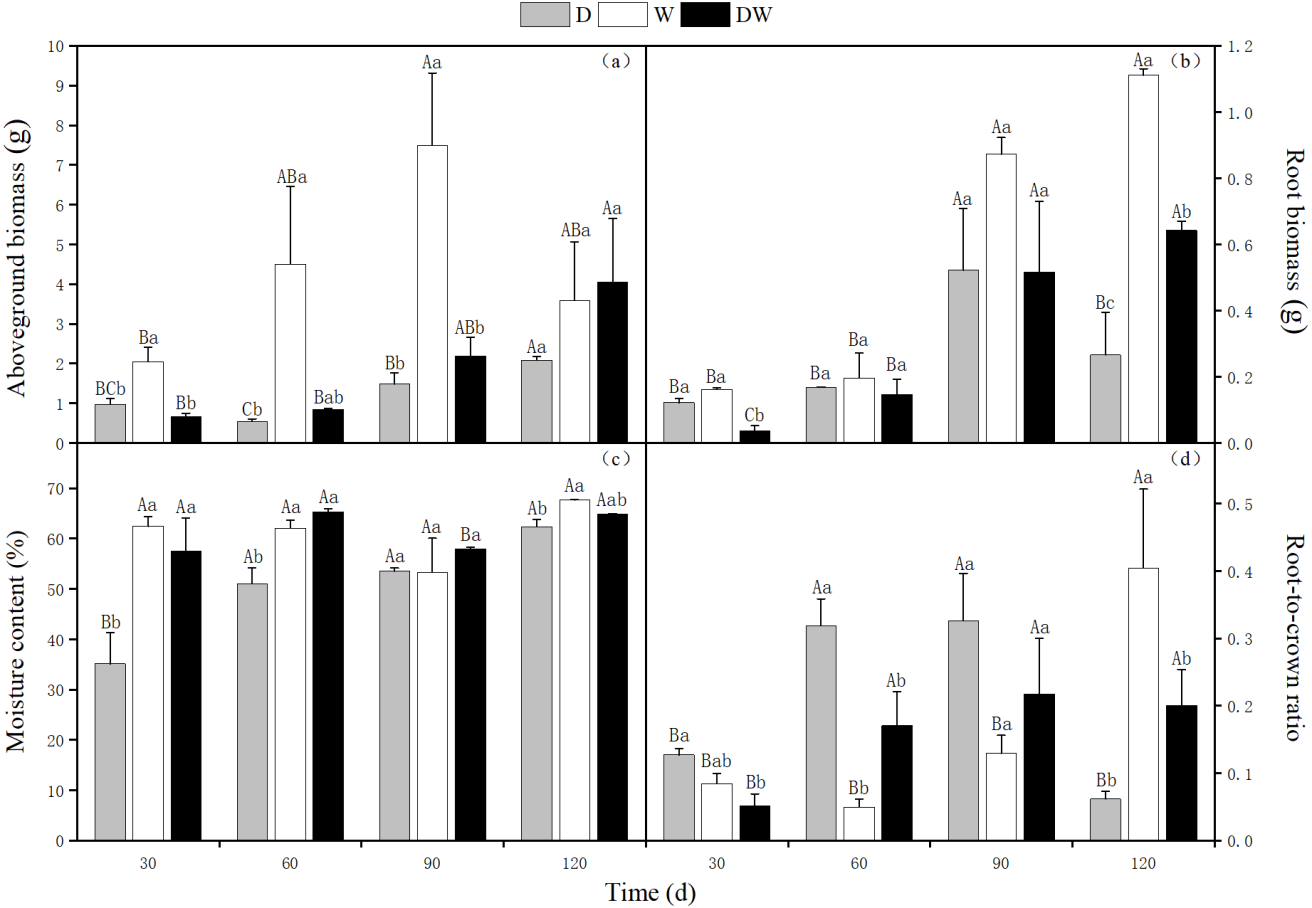
Effects of wet and dry changes on aboveground biomass, root biomass, moisture content and root-shoot ratio of *P. australis*. D, drought, W, permanent flooding, DW, alternating dry-wet. A,B,C, Differences between different times under the same water treatment. a,b,c, Differences between different water treatments at the same time. Different letters represent significant differences between the three treatments at the same time (*P* < 0.05), same letter means no significant difference (*P* > 0.05). Results are expressed as mean ± SE (*n* = 3).

The aboveground biomass of *P. australis* increased over time, reaching 2.08 and 4.05 g at 120 d under D and DW treatments, respectively. The weight was 12 and 6 times, respectively, being that at 30 d. The aboveground biomass showed a tendency of increasing first and then decreasing under W treatment, reaching a maximum value of 7.49 g at 90 d, which had slightly reduced at 120 d. The D, DW, and W treatments were significantly different at all sampling times except 120 d (Fig. 3A, *P* < 0.05).

The root biomass showed a trend of increasing first and then decreasing under D treatment, reaching a maximum of 0.52 g at 90 d. The root biomass showed an increasing trend, with up to 1.11 g and 0.64 g at 120 d, under W and DW treatments, respectively. The weight was 7 and 17 times higher than that at 30 d, with 0.16 and 0.04 g at 30 d. At 120 d, there were significant differences among the treatments (Fig. 3B, *P* < 0.05).

Under D treatment, the water content of *P. australis* shoots gradually increased with increasing drought period; at 120 d, it was 27.27% higher than at 30 d. The water contents under treatment W did not vary significantly among sampling periods. Although the water contents fluctuated alternately with drought and flooding under treatment DW, which was higher at 60 and 120 d, this difference was not significant. However, there were significant differences at all sampling times among treatment D, DW, and W, except for 90 d (Fig. 3C, *P* < 0.05). The water content was significantly higher under W and DW than under D (W > DW > D).

Under D and DW, root-to-crown ratio of *P. australis* first increased and then decreased, with the maximum values of 0.33 and 0.32, respectively, at 90 d. Due to sufficient water amounts, the aboveground parts grew more rapidly than the belowground ones. The root-to-crown ratio in treatment W decreased at 60 d. Later on, the root system thrived, and the root-to-crown ratio increased, with a maximum value of 0.40 at 120 d. The values differed significantly among D, DW, and W among all sampling times, except for 90 d (Fig. 3D, *P* < 0.05).

### *P. australis* root system characteristics

**Figure 4:**
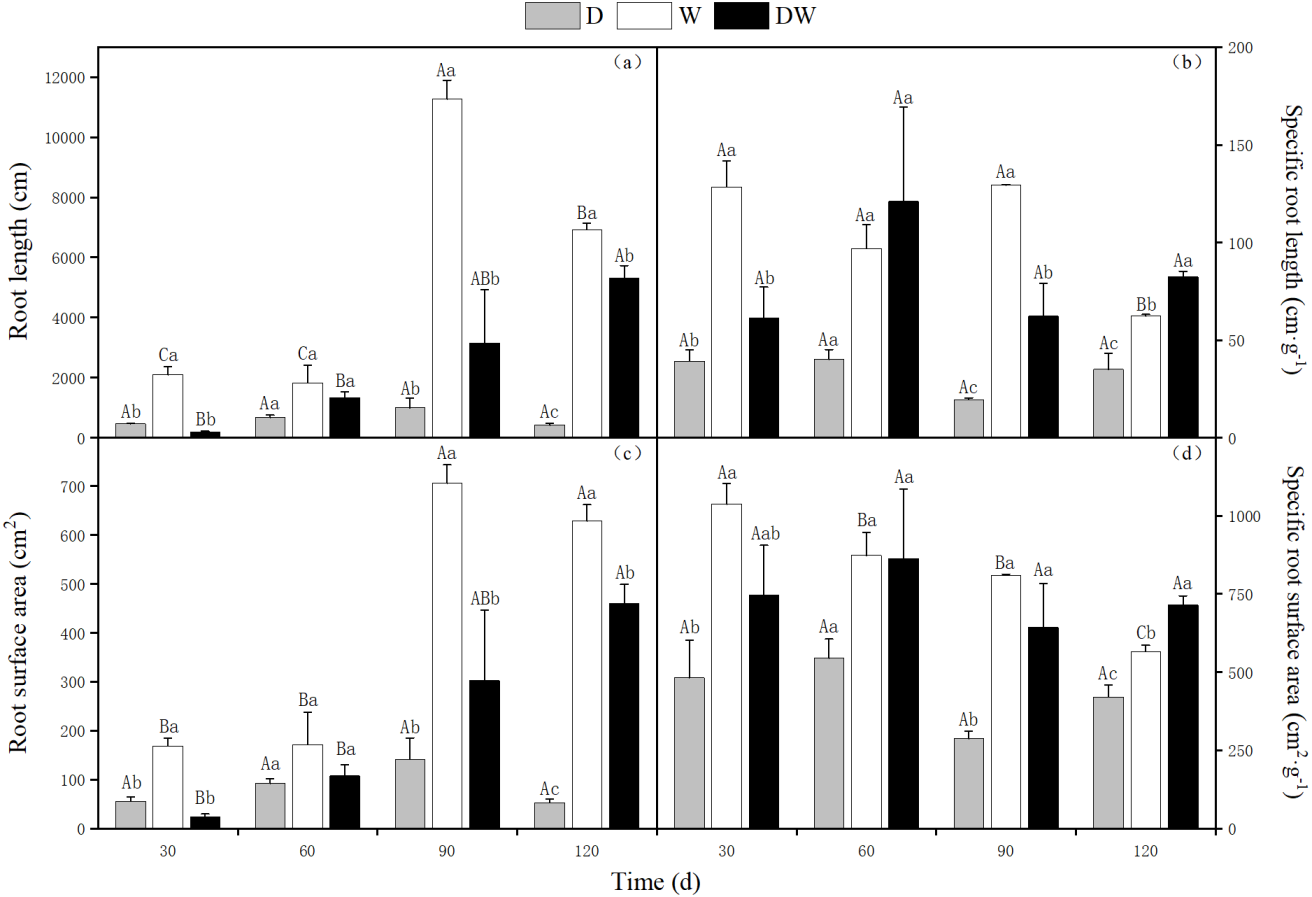
Effect of wet and dry changes on *P. australis* root system. D, drought; W, permanent flooding; DW, alternating dry-wet. A,B,C, Differences between different times under the same water treatment. a,b,c, Differences between different water treatments at the same time. Different letters represent significant differences between the three treatments at the same time (*P* < 0.05), same letter means no significant difference (*P* > 0.05). Results are expressed as mean ± SE (*n* = 3).

The lowest total and specific root length, root surface area, and specific root surface area values of *P. australis* were found under treatment D. The ratios of root length to particular root length were 16.5:12.6:1, and 1.8:2.3:1 for treatments W, DW, and D, respectively. The ratios between the root surface area and the specific surface area under W, DW, and D were 12:8.8:1, and 1.3:1.7:1, respectively. For treatment W, the highest root length and root surface area values were observed, whereas under DW, the highest specific root length and specific root surface area values were found. There were significant differences among the three hydrological treatments at all sampling times, except at 60 d (Fig. 4, *P* < 0.05).

**Figure 5:**
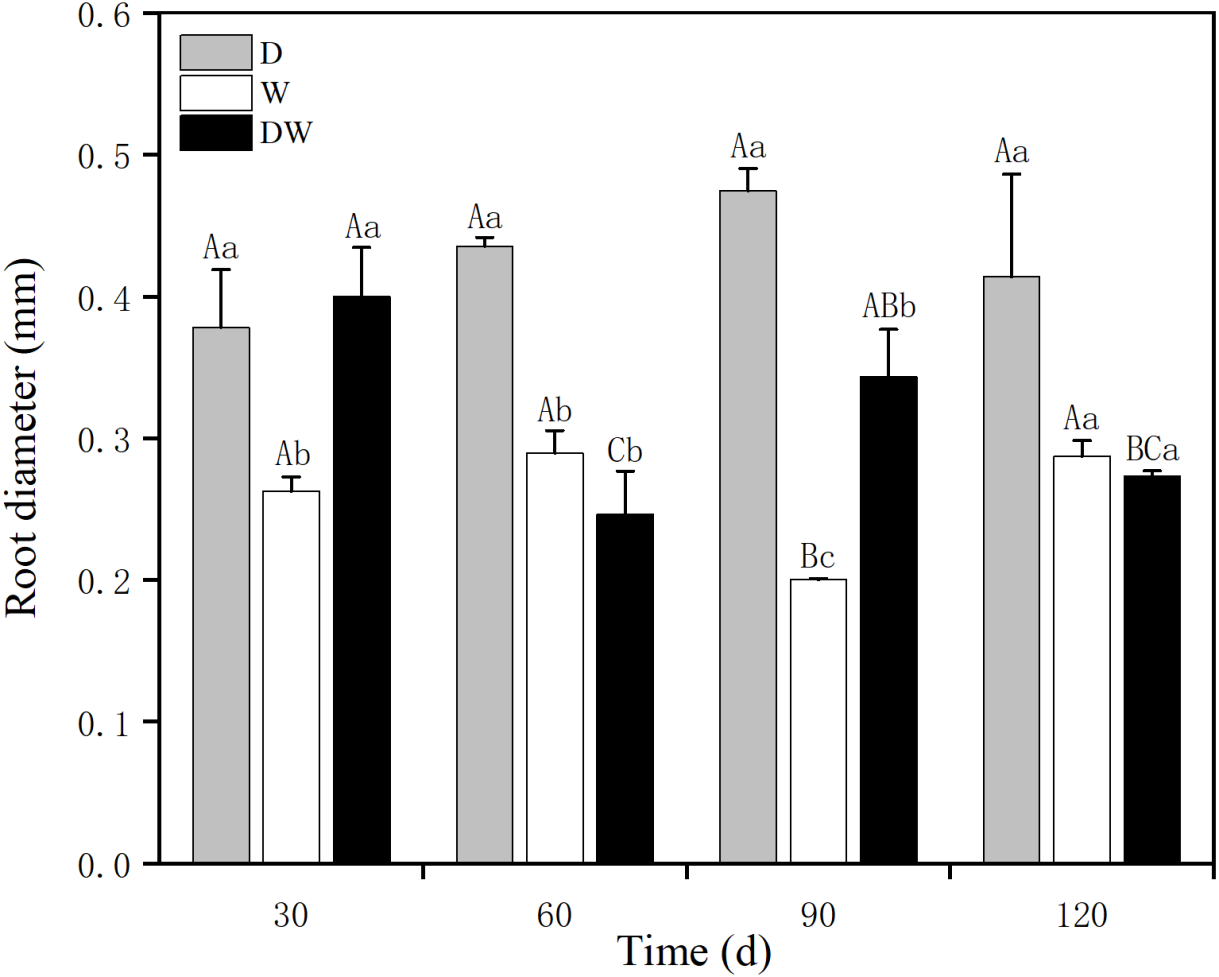
Effect of wet and dry variation on *P. australis* root diameter. D, drought; W, permanent flooding; DW, alternating dry-wet. a,b,c, Differences between different water treatments at the same time. Different letters represent significant differences between the three treatments at the same time (*P* < 0.05), same letter means no significant difference (*P* > 0.05). Results are expressed as mean ± SE (*n* = 3).

The *P. australis* root diameter ranged from 0.2–0.3 mm under W and from 0.3–0.5 mm under D, with mean values of 0.4 and 0.3 mm during the drought period and 0.25 and 0.27 mm during the flood period under DW, within the fluctuating range of the drought and flooding treatments. The values under all three hydrological treatments were significantly different at all sampling times, except at 120 d (Fig. 5, *P* < 0.05). Root diameter under D was substantially higher than under W and DW, following the order D > DW > W.

### Number of *P. australis* rhizome shoots and tiller seedlings

**Figure 6:**
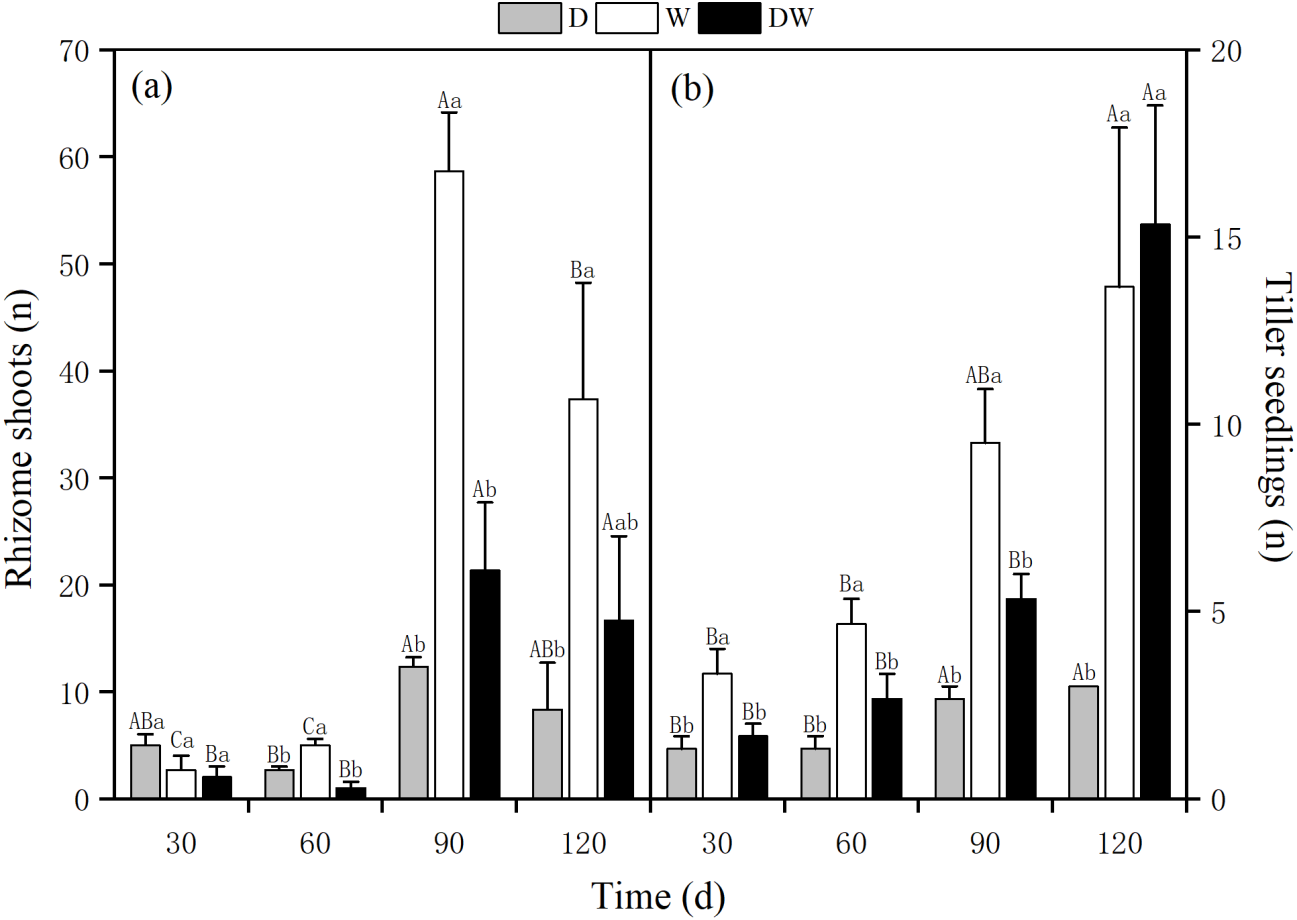
Effect of wet and dry changes on *P. australis* rhizome shoots and tiller seedlings. D, drought; W, permanent flooding; DW, alternating dry-wet. a,b,c, Differences between different water treatments at the same time. Different letters represent significant differences between the three treatments at the same time (*P* < 0.05), same letter means no significant difference (*P* > 0.05). Results are expressed as mean ± SE (*n* = 3).

The number of rhizome shoots of *P. australis* under each treatment had increased significantly at 90 d, with 12, 59, and 21, and showed a decreasing trend at 120 d for treatments 8, 37, and 17, respectively. The number of rhizome shoots under treatment W was markedly higher than under D and DW. Significant differences were found among the three hydrological treatments at all sampling times, except at 30 d (Fig. 6A, *P* < 0.05). The number of tiller seedlings of *P. australis* under all treatments had increased significantly, with the numbers of plant tiller seedlings under each treatment at 120 d being 3, 13, and 15. The number of tiller seedlings under W and DW was significantly higher than that under D. There was a significant difference in the number of tiller seedlings among all four sampling periods (Fig. 6B, *P* < 0.05).

### Photosynthetic physiology of *P. australis*

**Figure 7:**
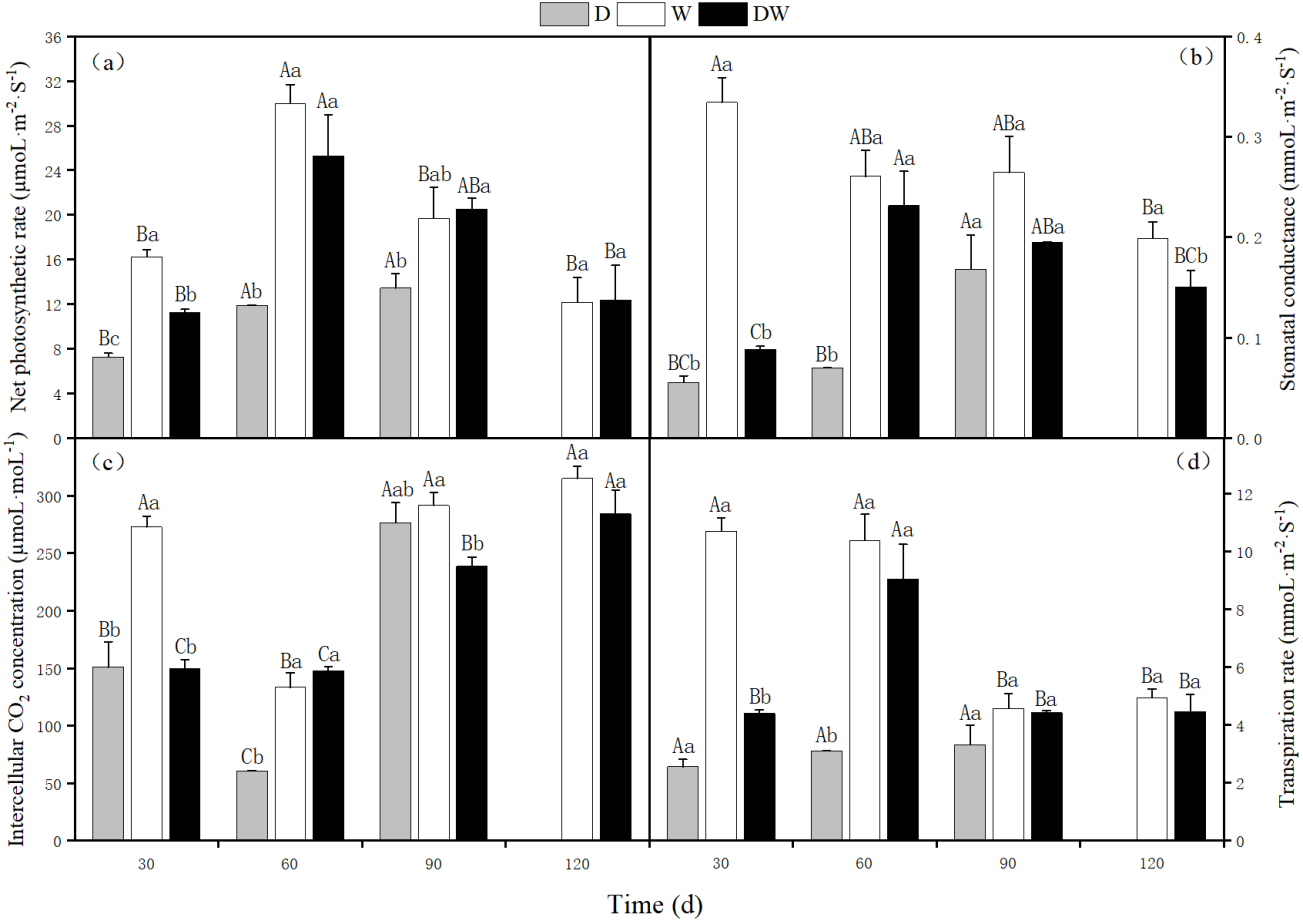
Effects of wet and dry changes on photosynthetic physiology of *P. australis*. D, drought; W, permanent flooding; DW, alternating dry-wet. A,B,C, Differences between different times under the same water treatment. a,b,c, Differences between different water treatments at the same time. Different letters represent significant differences between the three treatments at the same time (*P* < 0.05), same letter means no significant difference (*P* > 0.05). Results are expressed as mean ± SE (*n* = 3).

The net photosynthetic rate, stomatal conductance, intercellular CO_2_ concentration, and transpiration rate of *P. australis* were lowest under D, and at 120 d, the stems and leaves had yellowed from drought stress. The photosynthetic rate under treatment W first increased and then decreased, with a maximum at 60 d. Intercellular CO_2_ concentration showed the opposite trend, whilst stomatal conductance and transpiration rate showed a decreasing trend. Under DW, photosynthetic rate, stomatal conductance, and transpiration rate first increased and then decreased, with maximum values at 60 d. In contrast, the intercellular CO_2_ concentration increased (Fig. 7C). Net photosynthetic rate, intercellular CO_2_ concentration, and transpiration rate did not differ significantly among the three hydrological treatments at 30 and 60 d, whereas stomatal conductance differed significantly, except at 90 d (Fig. 7, *P* < 0.05).

### Metabolic physiology of *P. australis*

#### Soluble sugars

**Figure 8:**
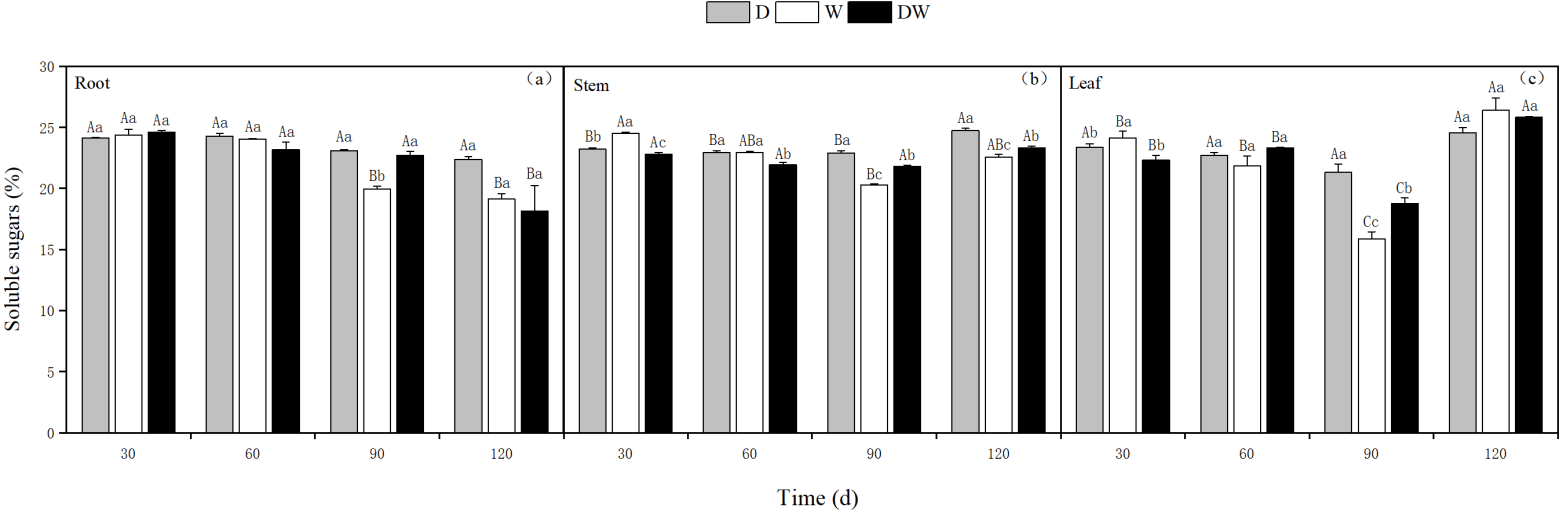
Effect of wet and dry changes on the soluble sugar content of *P. australis* roots, stems and leaves. D, drought; W, permanent flooding; DW, alternating dry-wet. A,B,C, Differences between different times under the same water treatment. a,b,c, Differences between different water treatments at the same time. Different letters represent significant differences between the three treatments at the same time (*P* < 0.05), same letter means no significant difference (*P* > 0.05). Results are expressed as mean ± SE (*n* = 3).

Under treatment D, the soluble sugar content in the roots decreased over time, whereas in the stems and leaves, it was relatively stable, with the highest levels at 120 d. Treatments W and DW showed a decreasing trend in soluble sugar content in roots, whereas in stems and leaves, the contents first decreased and then increased. The soluble sugar content in the roots was only significantly different at 90 d, whereas in the stems, it was significantly different at different sampling periods and in leaves at 30 and 90 d (Fig. 8, *P* < 0.05).

#### Proline

**Figure 9:**
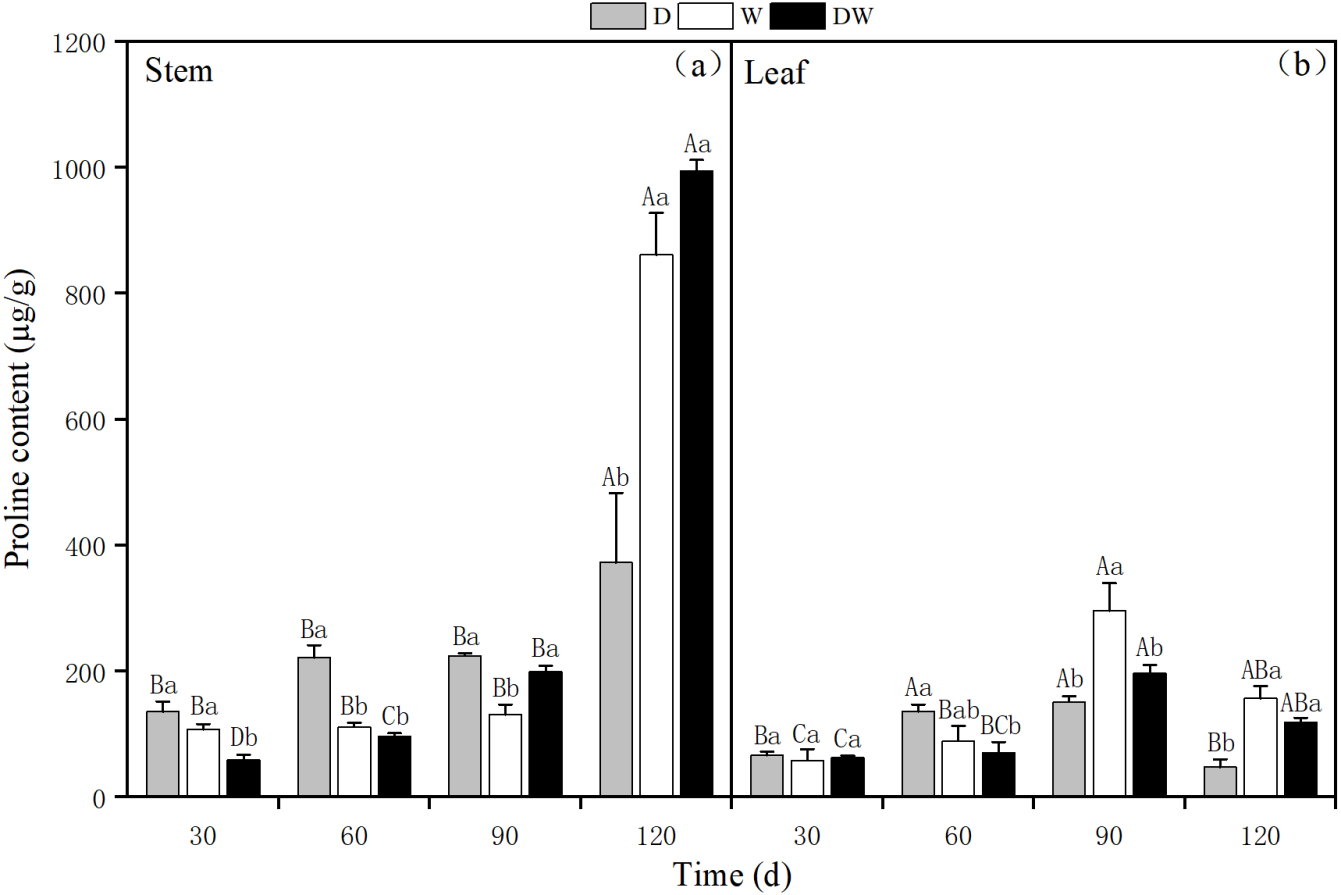
The effect of wet and dry changes on the proline content of *P. australis* stems and leaves. D, drought; W, permanent flooding; DW, alternating dry-wet. A,B,C, Differences between different times under the same water treatment. a,b,c, Differences between different water treatments at the same time. Different letters represent significant differences between the three treatments at the same time (*P* < 0.05), same letter means no significant difference (*P* > 0.05). Results are expressed as mean ± SE (*n* = 3).

The proline content in *P. australis* stems increased over time under all treatments, with 372.30, 861.39, and 994.24 µg g^−1^ under D, W, and DW at 120 d. During the middle and early stages of *P. australis* growth, the average proline content in stems under treatment D was higher than those under W and DW, in contrast to the trend at the end of the growth period for 120 d. Under W and DW, the proline content was significantly higher than under D; the stem proline content significantly differed among all four sampling periods (Fig. 9A, *P* < 0.05).

The proline content in *P. australis* leaves first increased and then decreased over time, irrespective of the treatment, with the highest levels at 90 d, namely 151.13, 295.81, and 197.14 µg g^−1^ under D, W, and DW. The proline content in leaves significantly differed among the different treatments, except at 30 d (Fig. 9B, *P* < 0.05). There was a significant difference in the proline content between stems and leaves under treatment D (*P* < 0.05), and the proline content in stems was significantly greater than that in leaves. In addition, under W and DW, the proline content was higher in stems than in leaves, although this difference was not statistically significant (Table 4, *P* > 0.05).

**Table 4:** Significance analysis of the mean values of stem and leaf proline content in the four periods of each treatment (Independent samples *t*-test).

**Treatment**	**Group**	**Proline (ug/g)**	
		**Average**	**Significance**
D	Root	238.4183^a^	0.047^*^
	Leaf	100.2064^b^	
W	Root	302.7346^a^	ns
	Leaf	149.9877^a^	
DW	Root	337.0932^a^	ns
	Leaf	112.3547^a^	

**Notes.**

a,b Difference of proline content in stems and leaves under the same treatment.

* Significantly different at *p* < 0.05.

#### Salt ion content

**Figure 10:**
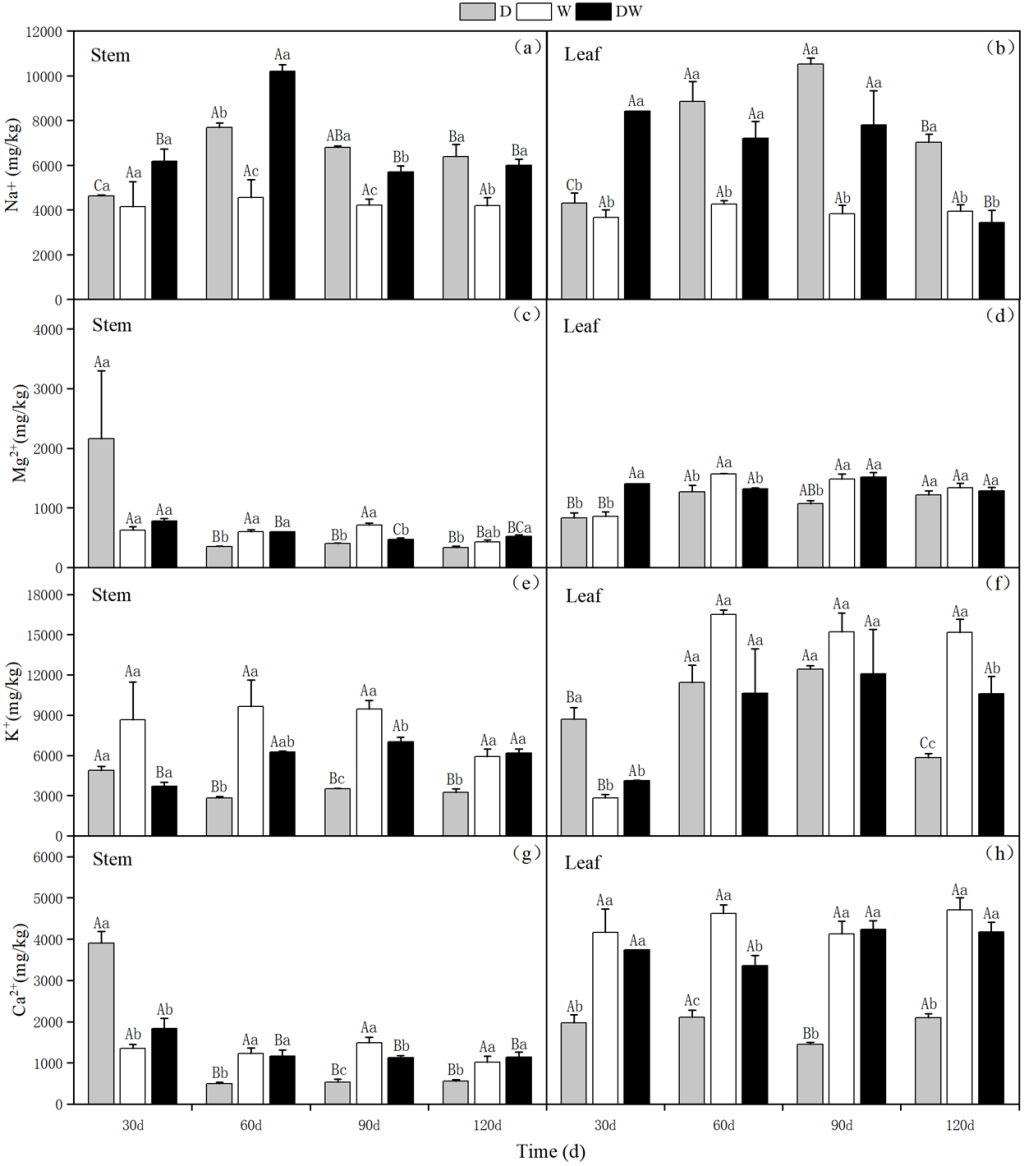
(A–H) Effect of wet and dry changes on the cation content of *P. australis* stems and leaves. D, drought; W, permanent flooding; DW, alternating dry-wet. A,B,C, Differences between different times under the same water treatment. a,b,c, Differences between different water treatments at the same time. Different letters represent significant differences between the three treatments at the same time (*P* < 0.05), same letter means no significant difference (*P* > 0.05). Results are expressed as mean ± SE (*n* = 3).

Under treatment D, Na^+^ in stems and leaves showed an increasing trend with increasing drought stress, with the highest levels in the stems and leaves at 60 and 90 d, respectively. Under DW, the Na^+^ content in stems increased and then decreased after rehydration, with the highest range at 60 d; however, in leaves, the Na^+^ content showed a decreasing trend. Under treatment W, the Na^+^ content in *P. australis* stems and leaves was low and stable during different periods because of the constant flooding. Under D and DW, the Na^+^ content in *P. australis* stems and leaves was higher than under W at different sampling periods, except for the stem Na^+^ content at 30 d, which did not differ significantly among the hydrological treatments (Figs. 10A and 10B, *P* < 0.05). There were no significant differences in the Na^+^ content in stems and leaves under the different treatments (Table 5).

**Table 5:** Significance analysis of the mean values of stem and leaf cation content in four periods for each treatment (Independent samples *t*-test).

**Treatment**	**Group**	**Na+ (mg/kg)**		**Mg2+ (mg/kg)**		**K+ (mg/kg)**		**Ca2+ (mg/kg)**	
		**Average**	**Significance**	**Average**	**Significance**	**Average**	**Significance**	**Average**	**Significance**
D	Root	6360.82^a^	Ns	812.427^a^	ns	3610.247^b^	0.023^*^	1371.422^a^	ns
	Leaf	7674.282^a^		1097.023^a^		9586.74^a^		1905.305^a^	
W	Root	4271.369^a^	Ns	590.328^b^	0.005^**^	8405.668^a^	ns	1270.468^b^	0.000^***^
	Leaf	3917.863^a^		1313.518^a^		12420.37^a^		4404.177^a^	
DW	Root	7019.351^a^	Ns	1610.253^a^	ns	5785.326^a^	ns	1316.973^b^	0.000^***^
	Leaf	6702.852^a^		1379.839^a^		9350.701^a^		3877.631^a^	

**Notes.**

a,b Difference of ion content in stems and leaves under the same treatment.

* Significantly different at *p* < 0.05.

** Significantly different at *p* < 0.01.

*** Significantly different at *p* < 0.001.

Under D and W, the K^+^ content in the stems tended to decrease, whereas under DW, it increased slightly and then decreased. The K^+^ content in the stems tended to decrease substantially with *P. australis* growth. Under D, the leaf K^+^ content first increased and then decreased. In contrast, under W and DW, the K^+^ content in the leaves increased substantially over time and then remained stable, with higher values under W than under DW. Significant differences in the K^+^ content in stems at 60 and 120 d and in leaves at 30 and 120 d were observed among the different treatments (Figs. 10E and 10F, *P* < 0.05). There was a significant difference in the K^+^ content in stems and leaves under D, following the order leaves >stems (Table 5).

Under D, the Ca^2+^ and Mg^2+^content in the stems was higher in the early stages of *P. australis* growth and then decreased substantially over time. The Ca^2+^ and Mg^2+^ contents in *P. australis* leaves under D and in stems and leaves under W and DW varied to a lesser extent among the sampling periods; it differed significantly among the sampling periods under different hydrological treatments. Under W and DW, *P. australis* stems and leaves showed significantly higher Ca^2+^ and Mg^2+^ contents than under D, except for the stem values at 30 d (Figs. 10C, 10D, 10G and 10H, *P* < 0.05). The trend of Mg^2+^ in *P. australis* stems and leaves under different hydrological treatments and at different sampling periods was similar to that of Ca^2+^. There was a significant difference in the Ca^2+^ content in stems and leaves under W and DW, following the order leaves >stems (Table 5). There was a significant difference in the Mg^2+^ content between stems and leaves under W, following the order leaves >stems (Table 5).

## Discussion

In wetland ecosystems, *P. australis* is a typical emergent plant and is significantly affected by hydrological changes. Under drought conditions, *P. australis* biomass was low, with less rhizome shoots and tiller seedlings, significantly inhibiting *P. australis* growth. In contrast, under flooding, biomass as well as the number of rhizome shoots and tiller seedlings of *P. australis* increased significantly, and the water content of stems and leaves stabilized, indicating that a stable flooded environment is more conducive to *P. australis* growth (Hellings & Gallagher, 1992). Alternating wet and dry conditions had no significant effect on *P. australis* biomass as well as rhizome shoots, and tiller seedlings during the first 2 months, but during the second 2 months, hydrological changes significantly promoted rhizome growth, with substantially higher biomass and more rhizome shoots and tiller seedlings, as well as an increased stem and leaf water content at each rewatering.

Based on the findings for the three treatments, it can be concluded that under flooding, *P. australis* are more capable of growing. According to a previous study, in saline soil environments, flooding does not increase the plants’ ability to bind water but reduces soil salinity to a certain extent (Baggie et al., 2018), creating good conditions for plants to survive. Therefore, when the *P. australis* is in a saline environment, flooding will make the *P. australis* grow vigorously. For *P. australis*, which has a sizeable belowground biomass, flood conditions are also more suitable for the growth of rhizome shoots, giving shoots the potential to form a rhizome or tiller plant and expand the underground shoot bank for a greater reproductive capacity (Guan et al., 2017).

Of all plant organs, the root system responds first to changes in soil moisture (Kato et al., 2007). This study found that drought significantly affected the *P. australis* root system in saline soil environments. Root biomass was lowest under drought stress, the diameter of the root system increased significantly, and the total root length and root surface area decreased substantially, which may be related to the increased salinization and intricate texture of the soil under drought conditions (Lissner & Schierup, 1997), impeding root extension. Hence, *P. australis* adapts to drought and saline conditions by allowing the root system to thicken, increasing the root-to-crown ratio. Flooding had the opposite effect, resulting in a lower root diameter, a significantly higher total length and root surface area, and a higher root biomass, along with an increased root-to-crown ratio. This indicates that *P. australis* roots grow more vigorously under flood conditions, improving the uptake and use of soil resources. In a previous study, under flooding, soil isolation from air led to a low oxygen content and reduced nutrient uptake and metabolism in some plants (Sauter, 2013); however, in the case of *P. australis*, a wetland plant, an anaerobic environment produces an extensive root system to enhance nutrient uptake (Tshapa, Naidoo & Naidoo, 2021). Under alternating wet and dry conditions, water control improved the drought conditions of saline soils. The root diameter of *P. australis* showed fluctuating changes with alternating wet and dry conditions, reflecting hydrological changes.

Specific root length and specific root surface area, as indicators of the root expansion capacity, directly influence the efficiency of soil nutrient uptake (Eissenstat, 1991). The specific root length and specific root surface area of the *P. australis* root system were significantly lower under drought conditions than under flooding and alternating wet and dry conditions, indicating that drought soil conditions and saline stress inhibited the ability of the root system to expand. In the alternating wet and dry treatment, each drought followed by flooding contributed to an increase in specific root length and specific root surface area, resulting in a significantly higher expansion capacity of the *P. australis* root system compared to the flood conditions. This indicates that *P. australis* can adapt to hydrological changes and can efficiently use water resources by regulating the growth and development of its root system.

The growth characteristics of *P. australis* populations under different hydrological treatments are closely related to photosynthetic and metabolic physiology. Net photosynthetic rate is a direct indicator of the reflected strength of photosynthesis in plants (Yu et al., 2017). In *P. australis* under drought conditions, the net photosynthetic rate was significantly lower than that under flooding and alternating wet and dry treatments, indicating that prolonged drought stress negatively impacts plant growth, resulting in leaf yellowing and damage to the photosynthetic organs, which in turn affects photosynthesis in the later stages of growth. The differences in net photosynthetic rate and transpiration rate between the wet and dry alternation treatments and the flooding treatment were not significant, indicating that *P. australis* achieves positive feedback regulation under short-term drought by regulating its allocation strategy; this verifies that wet and dry alternation can replace long-term flooding to restore the aboveground biomass of the first generation of *P. australis*. It has been suggested that reduced photosynthetic rates may also be associated with reduced stomatal conductance and that stomata play a crucial role in water regulation (Yamori et al., 2020). Under drought stress, plants overcome water imbalance by regulating stomatal closure (Martin-StPaul, Delzon & Cochard, 2017). The reduction in stomatal conductance also affects the intercellular CO_2_ transport (Tezara et al., 2002), thus exerting an inhibitory effect on photosynthesis, which is consistent with the results of this study. According to a previous study, *P. australis* often suffers from physiological “drought” under saline stress, where stomata close and transpiration decreases, resulting in insufficient carbon accumulation and impeded growth and development (Yang, Xie & Liu, 2014).

When subjected to drought stress, plants usually increase their soluble sugar and proline contents to enhance stress resistance (Li et al., 2014a; Li et al., 2014b). In this study, the soluble sugar content in *P. australis* stems and leaves was significantly higher under drought conditions than under flooding and alternating wet and dry treatments. Proline is an essential osmoregulatory organic solute in plants and can be used as an osmoregulatory substance to maintain water balance; it also participates in the synthesis of chlorophyll, is an antidote to plant ammonia, and has a strong hydration capacity (Ashraf & Foolad, 2007). It is generally accepted that the proline content is greater under drought conditions than under flooded conditions(Verbruggen & Hermans, 2008), but in this study, the opposite was observed. The proline content in the drought treatment was higher than that of the alternating wet and dry treatment during the early growth period, but in the late growth period, the proline content of the alternating wet and dry treatment was considerably higher than that of the drought treatment. This can be explained by the accumulation of proline as an osmoregulatory response induced by a period of inundation. On the other hand, it may also be a collective response of the plant’s cell structure and function when it is damaged by continuous inundation (Zheng et al., 2020). The proline content of *P. australis* stems was much higher than that of leaves, most likely because saline stress affects the accumulation of proline in the leaves, causing them to produce less proline. It may also be due to the increased K^+^, Ca^2+^, and Mg^2+^ contents in the root system, reducing proline accumulation in the leaves (Yang et al., 2018).

Under drought conditions, plants take up more Na^+^ than K^+^, which plays a more important role in osmoregulation than K^+^, and Na^+^ accumulates mainly in the leaves of plants, playing an osmoregulatory role (Li et al., 2014a; Li et al., 2014b). In this study, Since the study was selected from a saline marsh, *P. australis* were significantly stressed by salinity under drought conditions in the three hydrological treatments. The Na^+^ contents in *P. australis* stems and leaves in saline environments were high at all four sampling times since drought conditions, and Na^+^ was involved in the osmoregulation of *P. australis* stems and leaves under drought conditions. Some studies have pointed out that although K^+^ can alleviate the degree of stress under saline conditions, high concentrations of Na^+^ can affect the uptake of K^+^ by plants (Wakeel et al., 2011). It is also evident from the results of this experiment that the K^+^ content in *P. australis* stems and leaves is extremely low when the Na^+^ content is high under drought conditions and that the Na^+^ content in *P. australis* stems and leaves during flooding is low and the K^+^ content is high. The Na^+^ content of the stems and leaves also decreased after each rehydration under alternating wet and dry treatments, accompanied by an increase in the K^+^ content. This demonstrates that flooding reduces the Na^+^ content and increases the K^+^ content, facilitating plant growth under saline conditions. Under drought conditions, Ca^2+^ content in *P. australis* stems decreases with increasing time. This is because various physiological changes occur in plants under drought stress conditions, and Ca^2+^ uptake by the root system is inhibited, mainly due to the reduced ability of the root system to absorb Ca^2+^ from the growing environment, which can cause Ca^2+^ deficiency in the plant and consequently produce various adverse reactions (Li, Wang & Cheng, 2019). The reason for the decrease in Mg^2+^ content in *P. australis* stems under drought is that drought causes the cell walls of the plant to break down, resulting in the loss of Mg^2+^ (Pei, Zhang & Gao, 2001).

Overall, although continuous flooding promoted *P. australis* growth, alternating wet and dry was more beneficial to *P. australis* growth than continuous flooding. The expansion ability of *P. australis* roots under alternating wet and dry treatment was the strongest, which facilitated the recovery of *P. australis* populations. It also provides a favorable method for *P. australis* population recovery in the context of water conservation.

## Conclusions

The above- and belowground parts of *P. australis* showed different responses to hydrological changes, and both flooding and alternating wet and dry treatments could effectively improve the growth, reproduction, and physiological functions of *P. australis*. When the habitat is unsuitable, *P. australis* enters a latent state, and when waterlogging occurs, its growth capacity became stronger. Both flooding and alternating wet and dry treatment can be used to effectively restore *P. australis* population density (tillering) and biomass after drought. However, the alternation of wet and dry conditions uses water more efficiently and meets the water needs of *P. australis*, promoting rooting, increasing biomass and the numbers of rhizome shoots and tiller seedlings, improving the photosynthetic capacity, and facilitating the recovery of *P. australis* saline marshes. However, in terms of differences in root and rhizome shoot numbers, alternating wet and dry conditions can replace prolonged inundation and restore the aboveground productivity of *P. australis* in the current season, but has insufficient potential for *P. australis* population growth in the next growing season (Li et al., 2015). Therefore, for the recovery of *P. australis* populations on heavily saline soils, full season irrigation is more suitable when sufficient water is available, increasing the density of *P. australis* in the following year.

##  Supplemental Information

10.7717/peerj.14269/supp-1 Data S1 Raw data

## Additional Information and Declarations

### Data Availability

The following information was supplied regarding data availability: The raw measurements are available as Supplemental File.
